# CREATING OPPORTUNITIES TO IMPROVE AGE INCLUSIVITY IN HIGHER EDUCATION: AN INNOVATIVE EDUCATION PROGRAM EXAMPLE

**DOI:** 10.1093/geroni/igae098.1433

**Published:** 2024-12-31

**Authors:** Michelle Porter

**Affiliations:** University of Manitoba, Winnipeg, Manitoba, Canada

## Abstract

The Centre on Aging at the University of Manitoba (UM) leads the UM Age-friendly University (AFU) Committee and has taken on many initiatives to enhance the age inclusivity of the University, since 2016. In 2021, the Centre held a competition called the Age-friendly University Initiative Fund, which was open to all academic and non-academic units at UM. Funded projects related to campus wayfinding (Architectural & Engineering Services and the Office of Sustainability), inter-generational arts presentations/workshops (School of Art Gallery), technology training (Alumni Relations), and the development of a micro-certificate on Facilitating Older Adult Learning (FOAL; Faculty of Extended Education). This presentation will provide an overview of the Age-friendly University Initiative Fund and its collaborative process of working with funded projects, as well as provide specific details on the development and implementation of the micro-certificate. The FOAL micro-certificate provides a professional development opportunity to take three modules online: older adult development, universal design for learning, and using technology for teaching and learning with older adults. In total there are 36 contact hours for this credential that can be completed in a flexible fashion. The goal of the micro-certificate is to enable individuals to work more effectively with older adults to support their learning, across a broad range of learning environments (e.g., lifelong learning programs, senior centers, health care and therapy settings). Cross-unit collaboration is critical for the effective development and implementation of initiatives to improve age inclusivity across multiple domains such as those described in this presentation.

